# Real-world effectiveness of sucroferric oxyhydroxide in patients on chronic hemodialysis: A retrospective analysis of pharmacy data 

**DOI:** 10.5414/CN109021

**Published:** 2017-06-07

**Authors:** Daniel W. Coyne, Linda H. Ficociello, Vidhya Parameswaran, Ludmila Anderson, Sharanya Vemula, Norma J. Ofsthun, Claudy Mullon, Franklin W. Maddux, Robert J. Kossmann, Stuart M. Sprague

**Affiliations:** 1 Washington University School of Medicine, St. Louis, MO,; 2 Fresenius Medical Care North America, Waltham, MA, and; 3 NorthShore University Health System-University of Chicago, Pritzker School of Medicine, Evanston, IL, USA

**Keywords:** sucroferric oxyhydroxide, pill burden, phosphorus

## Abstract

Aims: Hyperphosphatemia has been associated with an increased risk of mortality in patients with end-stage renal disease. We sought to assess the real-world effectiveness of sucroferric oxyhydroxide (SO), an iron-based phosphate binder (PB), in control of serum phosphorus levels, and to determine the associated pill burden in hemodialysis patients. Materials and methods: Adult, in-center hemodialysis patients first prescribed SO through a renal pharmacy service as part of routine clinical care between April 1, 2014 and March 31, 2015 were included in the analysis. The proportion of patients with phosphorus levels ≤ 5.5 mg/dL and the mean prescribed PB pills/day were compared between baseline (3 months prior to SO) and SO follow-up at 3 (SO 1 – 3) and 6 months (SO 4 – 6). Mineral bone disease markers, hemoglobin, iron indices, and erythropoiesis-stimulating agents and intravenous iron use were assessed. Results: At baseline, all patients (n = 1,029) were prescribed PB, and 13.9% had mean serum phosphorus ≤ 5.5 mg/dL. Comparing baseline to SO 1 – 3, the mean prescribed PB pills/day declined from 9.6 to 3.8 pills/day (p < 0.001), and the proportion of patients with serum phosphorus ≤ 5.5 mg/dL increased from 13.9 to 26.1% (+88%). Comparing baseline to SO 4 – 6 (n = 424), the mean prescribed PB pills/day declined from 9.7 to 4.0 pills/day (p < 0.001), and the proportion of patients with serum phosphorus ≤ 5.5 mg/dL increased from 15.6 to 30.4% (+95%). Conclusions: Prescription of SO was associated with an increase in the proportion of patients achieving serum phosphorus levels ≤ 5.5 mg/dL along with fewer prescribed PB pills/day.

## Introduction 

Serum phosphorus above the National Kidney Foundation Kidney Disease Outcomes Quality Initiative (KDOQI) recommend range of 3.5 – 5.5 mg/dL has been associated with increased mortality in dialysis patients [1, 2]. Phosphorus control is sought through hemodialysis (HD), dietary restriction, and use of phosphate binders (PB) [2]. Despite these efforts, 37% of HD patients in the United States have serum phosphorus exceeding 5.5 mg/dL [3]. 

A high daily PB pill burden is a recognized barrier to treatment adherence [4, 5, 6]. Patients on HD have a high overall pill burden (on average 19 pills/day) with half of the pills attributed to PB [4]. A recent Dialysis Outcomes and Practice Patterns Study publication reported that, in the United States, fewer than half of patients reported taking all prescribed PBs in the prior month [6]. PB adherence has been inversely associated with phosphorus levels [7]. 

Sucroferric oxyhydroxide (SO, Velphoro, Fresenius Medical Care Renal Therapies Group, Waltham, MA, USA) is an iron-based, chewable PB indicated for treatment of hyperphosphatemia in patients with chronic kidney disease on dialysis. A phase III study demonstrated non-inferiority of SO to sevelamer (Sev) in serum phosphorus reduction [8]. Over 52 weeks in the phase III extension study, the mean number of SO pills taken was 3.3 compared with 8.7 pills/day for Sev [9]. HD patients who volunteer and adhere to a clinical trial protocol may be different from the general population of HD patients. In order to assess the real-world effectiveness of SO, serum phosphorus levels, mean prescribed number of pills per day, and other clinical parameters were compared at baseline, and following 3 and 6 months of SO treatment in adult, in-center HD patients prescribed SO as part of routine clinical care. 

## Materials and methods 

De-identified patient and laboratory data were retrospectively extracted from the Fresenius Kidney Care (FKC) electronic records. De-identified prescription fill information was extracted from the electronic records of FreseniusRx, a specialty renal pharmacy service. Eligible FKC patients were at least 18 years old, received in-center HD, were previously prescribed PB, and received their first SO prescription between April 1, 2014 and March 31, 2015 through FreseniusRx. All prescriptions were made by the attending nephrologist as part of their routine clinical care. This analysis did not impact prescriptions of PB or prescribing patterns. Treatment intervals were defined as baseline (3 months prior to SO prescription), SO 1 – 3 (first 3 months of SO prescription), and SO 4 – 6 (4 – 6 months of SO prescription). Patients prescribed combination PB therapy (> 1 PB prescribed on same day) and patients with no recorded PB prescriptions at baseline were excluded. Due to lack of information about any remaining PB supply on hand at a patient’s home, new PB prescriptions were assumed to discontinue the prior PB prescription. Duration of prescription was calculated from date of fill and number of days supplied. Follow-up on SO ended when there was no SO for > 60 days or a switch to another PB. Out of the 1,029 patients with follow-up during SO 1 – 3, 605 patients were not included in the 4 – 6 month analysis. Reasons for end of SO follow-up before SO 4 – 6 were no PB fills at FreseniusRx or prescription of another PB. 

Basic demographic characteristics, HD vintage, and major comorbid conditions were evaluated. The first date of dialysis at a FKC was used to calculate dialysis vintage if date of first dialysis was unavailable. Additional clinical characteristics encompassed the mean prescribed number of pills, laboratory measures (albumin, hemoglobin, ferritin, transferrin saturation, phosphorus, calcium, intact PTH, equilibrated Kt/V (eKt/V), equilibrated normalized protein catabolic rate (enPCR), and anemia therapy medications (intravenous (IV) iron and erythropoiesis-stimulating agent (ESA)). All laboratory tests were standardized and performed by Spectra Laboratories (Rockleigh, NJ, USA), and results were automatically downloaded to the FKC database. The standard practice is to measure albumin, hemoglobin, transferrin saturation, phosphorus, calcium, eKt/V, and enPCR monthly and ferritin and intact PTH quarterly. All available laboratory measures were averaged over each treatment period. Values for quarterly labs, when unavailable for the treatment period, were substituted with the most recent value. 

Two approaches were used to analyze serum phosphorus levels. First, we assessed changes in the percent of patients with serum phosphorus at or below 5.5 mg/dL [6]. Next, we analyzed the percent of patients at 1 mg/dL increments of serum phosphorus, disregarding the 5.5 mg/dL threshold. Mean prescribed baseline and follow-up PB number of pills per day were calculated by weighted averages when pills per day changed over an interval, and estimates for number of pills per day > 20 were recorded as missing. 

Means and standard deviations and counts and proportions were used to describe continuous and categorical variables, respectively. Pair-wise comparisons of continuous data were carried out using paired t-test, and categorical data were compared using McNemar’s χ^2^-test. p-values < 0.05 were considered statistically significant. All analyses were conducted using SAS version 9.4 (SAS Institute Inc., Cary, NC, USA). 

This database study was approved by the New England Institutional Review Board. SO is commercialized in the United States by Fresenius Medical Care Renal Therapies Group. 

## Results 

### Cohort with 1 – 3 months of SO prescription 

Overall, 1,029 adult, in-center HD patients with recorded SO prescription orders were included. Patient demographics are presented in Table 1. 

**Figure 1.**
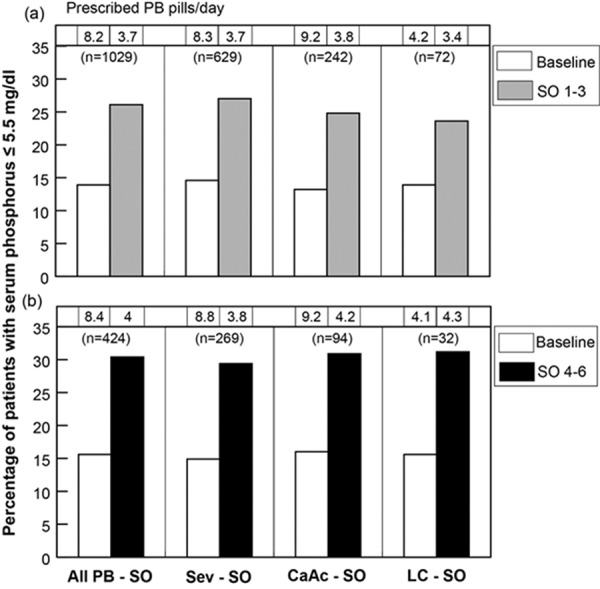
Change in percentage of patients with serum phosphorus ≤ 5.5 mg/dL switching from all phosphate binders (all PB), sevelamer (Sev), calcium acetate (CaAc), and lanthanum carbonate (LC) to sucroferric oxyhydrase (SO).

During SO 1 – 3, a decrease in mean number of PB pills (9.6 vs. 3.8 pills/day, p < 0.001) was observed (Table 2). An 88% increase in the proportion of patients achieving serum phosphorus ≤ 5.5 mg/dL (13.9% baseline vs. 26.1% SO 1 – 3, p < 0.001) was noted. Patients previously treated with Sev (former Sev patients) were prescribed on average 9.9 pills/day at baseline compared with 3.8 pills/day during SO 1 – 3. The proportion of former Sev patients with mean phosphorus ≤ 5.5 mg/dL increased from 14.6% at baseline to 27.0% during SO 1 – 3 (p < 0.001). Patients previously treated with calcium acetate (CaAc; former CaAc patients) were prescribed on average 10.6 pills/day at baseline compared with 3.8 pills/day during SO 1 – 3 (p < 0.001). The proportion of former CaAc patients with mean phosphorus ≤ 5.5 mg/dL increased from 13.2 to 24.8% during SO 1 – 3 (p < 0.001). Patients previously treated with lanthanum carbonate (LC; former LC patients) were prescribed on average 4.9 pills/day at baseline compared with 3.8 pills/day during SO 1 – 3 (p < 0.001). The proportion of former LC patients with mean phosphorus ≤ 5.5 mg/dL increased from 13.9 to 23.6% during SO 1 – 3 (p = 0.05) (Figure 1). 

**Figure 2.**
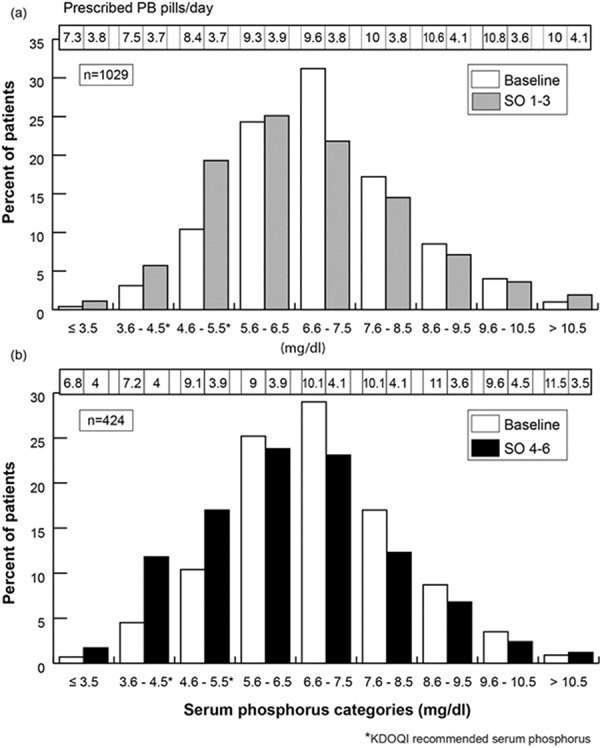
Change in distribution of patients across different serum phosphorus categories at baseline and during sucroferric oxyhydrase (SO) treatment.

Figure 2 depicts overall changes in distribution of patients across the serum phosphorus categories. During SO 1 – 3, the proportion of patients at or below 5.5 mg/dL increased while the proportion of patients between 5.6 and 10.5 mg/dL decreased. A minor increase was noted in patients above 10.5 mg/dL (1.0% vs. 1.9%; p = 0.06). Across all categories, the mean prescribed number of pills per day declined; for patients in categories above 3.5 mg/dL, the number of pills per day declined by more than 50%. 

The assessment of concomitant medications showed a decrease in the proportion of patients treated with any IV iron (from 90.5 to 80.4%; p < 0.001) and ESA (from 86.4 to 82.5%; p < 0.001). Epoetin-α dose per administration decreased from 5,225 to 4,957 units/administration (p = 0.01). In patients on IV iron therapy, transferrin saturation increased from baseline 34.1% to 35.5% at SO 1 – 3 (p = 0.001), while no changes in iron indices were observed for patients not on IV iron. Clearance and nutritional parameters changed minimally, with an exception of enPCR that decreased from 0.97 to 0.95 g/kg/day (p = 0.001) (Table 2). 

### Cohort with 4 – 6 months of SO prescription 

Overall, 424 adults were included in the analysis. Patient demographics are presented in Table 1. 

A decrease in mean number of PB pills per day (9.7 pills/day during baseline vs. 4.0 pills/day during SO 4 – 6, p < 0.001) was observed (Table 3). Between baseline and SO 4 – 6, there was a 95% increase in the proportion of patients achieving ≤ 5.5 mg/dL (15.6 to 30.4%, p < 0.001). Former Sev patients were prescribed, on average, 10.1 pills/day of Sev at baseline (p < 0.001) compared with 3.9 pills/day during SO 4 – 6. The proportion of former Sev patients with phosphorus level ≤ 5.5 mg/dL increased from 14.9 to 29.4% during SO 4 – 6 (p < 0.001). Former CaAc patients were prescribed, on average, 10.7 pills/day of CaAc at baseline compared with 4.0 pills/day during SO 4 – 6 (p < 0.001). The proportion of former CaAc patients with phosphorus level ≤ 5.5 mg/dL increased from 16.0 to 30.9% during SO 4 – 6 (p = 0.004). Former LC patients were prescribed on average 4.7 pills/day at baseline compared with 4.3 pills/day during SO 4 – 6 (p = 0.2). The proportion of former LC patients with phosphorus level ≤ 5.5 mg/dL increased from 15.6 to 31.2% during SO 4 – 6 (p = 0.06) (Figure 1). 

Table 3 shows changes in concomitant medications. The use of any IV iron significantly declined from 84.2 to 70.2% (p < 0.001), so did the use of any ESA (86.3 vs. 82.2%; p = 0.01). Significant changes were observed for IV iron sucrose dose per week (67.8 vs. 58.2 mg/week; p = 0.02), epoetin-α use (94.7 vs. 87.0%; p < 0.001), epoetin-α dose per HD treatment (3,613 vs. 3,182 units/treatment; p = 0.03), and epoetin-α dose per administration (5,397 vs. 4,805 units/administration; p = 0.02). Changes in clinical parameters for patients on IV iron therapy revealed that serum ferritin increased between baseline and SO 4 – 6 (984.1 to 1,070.7 ng/ml, p < 0.001), while transferrin saturation and hemoglobin did not change. In patients not treated with IV iron, serum ferritin decreased (1,161 to 926.5 ng/mL; p = 0.005) between baseline and SO 4 – 6, while hemoglobin and transferrin saturation stayed the same. Clearance and nutritional parameters changed minimally from baseline, with an exception of enPCR that changed from 0.96 to 0.94 g/kg/day (p = 0.01). 

Figure 2 depicts overall changes in distribution of patients with 4 – 6 months of follow-up across the serum phosphorus categories. At 6 months of follow-up, an increase in the proportion was noted for patients in categories ≤ 5.5 mg/dL and a simultaneous decrease in proportions above 5.5 mg/dL. Across all categories, the mean number of prescribed pills per day declined; in categories above 4.5 mg/dL, this decline was more than 50%. 

### Cohort not included in 4 – 6 month cohort 

**Figure 3.**
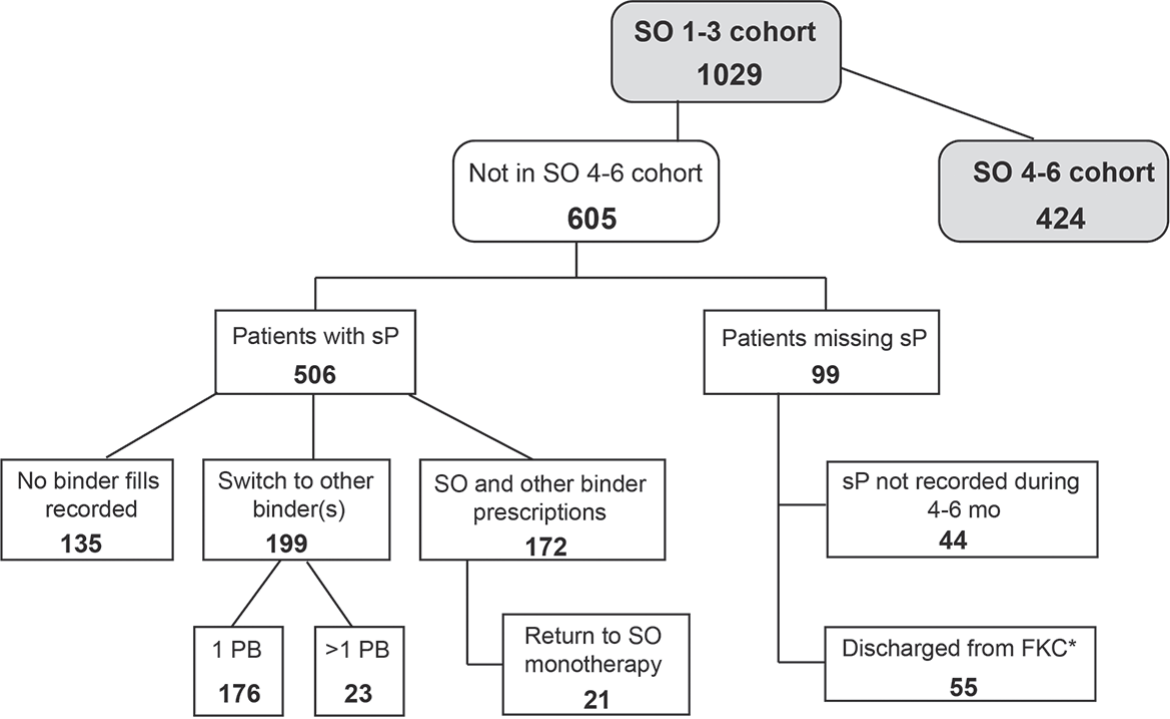
Patient disposition. The SO 1 – 3 cohort included 1,029 patients. The SO 4 – 6 cohort included 424 patients, and 605 patients were not included. Figure 3 shows the disposition of 605 patients not included in SO 4 – 6 cohort. *Discharge due to transfer, transplant, or death. FKC = Fresenius Kidney Care; PB = phosphate binder; SO = sucroferric oxyhydroxide; sP = serum phosphorus.

605 patients included in the 1 – 3 month cohort were not eligible for the 4 – 6 month cohort (Figure 3) because their serum phosphorus was missing (n = 99) or serum phosphorus was available, but patients had no PB prescriptions recorded at FreseniusRx (n = 135), had been switched to other PB or PBs (n = 199), or prescribed SO and other PB (n = 172). The 135 patients with no binder information at FreseniusRx may have received SO or another PB from a different pharmacy. Table 4 presents the PB therapy received at 4 – 6 months in the patients switched from SO (n = 199). The most frequent monotherapy switch was to sevelamer (n = 108) and the most frequent dual therapy switch was to calcium acetate and sevelamer (n = 14). There were 134 patients who switched back to the same binder that they were prescribed before they began SO, including 90 patients prescribed sevelamer, 41 prescribed calcium carbonate, and 3 lanthanum carbonate. 

During 4 – 6 months, the 135 patients with no PB recorded had mean serum phosphorus of 6.29 mg/dL with 31.9% of patients with serum phosphorus ≤ 5.5 mg/dL, the patients switched to another binder had a mean serum phosphorus of 6.47 mg/dL with 25.1% of patients with serum phosphorus ≤ 5.5 mg/dL, and the patients with prescription fills of SO and another binder had a mean serum phosphorus of 6.45 mg/dL with 25% of patients having serum phosphorus < 5.5 mg/dL. Of the patients with recorded PB, mean number of pills per day for patients on monotherapy was 9.8 ± 5.1 pills and on > 1 binder was 13.8 ± 5.8 pills. In comparison, the eligible 4 – 6 month cohort (n = 424) had a mean serum phosphorus of 6.41 mg/dL with 30.4% of patients with ≤ 5.5 mg/dL and average number of PB pills per day of 4.0 (Table 3). 

## Discussion 

A significant decline in the mean prescribed number of PB pills per day was observed among adult, in-center, chronic HD patients during 3 and 6 months of SO treatment. This decline was coupled with a significant increase in the proportion of patients with mean phosphorus ≤ 5.5 mg/dL. All patients included in the analysis were treated with PB at baseline; however, only 13.9% had serum phosphorus levels ≤ 5.5 mg/dL, which is substantially less than what has been reported in national data [3]. This would seem to indicate that SO was mainly prescribed to hyperphosphatemic, difficult-to-treat patients. Nevertheless, the proportion of patients achieving the recommended range of serum phosphorus increased 88% during SO 1 – 3 and 95% during SO 4 – 6 of treatment. Treatment non-adherence due to a high daily pill burden is a commonly cited barrier to hyperphosphatemia control [4]. The fact that the observed mean prescribed number of PB pills was reduced by more than 50% during the follow-up period is promising; particularly since this reduction occurred at 3 as well as at 6 months of follow-up. These findings of improved phosphorus control with lower pill number on SO treatment suggest that a lower pill burden may be associated with better PB adherence. Data was retrospectively collected, so there was no effect of the project on prescription adherence. We observed that the overall mean prescribed number of pills per day was 3.8 and 4.0 pills/day at 3 and 6 months of follow-up, respectively, including patients with serum phosphorus above 5.5 mg/dL. Perhaps an increase in prescribed SO daily pills for patients with phosphorus levels > 5.5 mg/dL could be considered. 

Similar results were reported previously by Floege et al. [8, 9], who reported that SO was as effective as Sev in lowering serum phosphorus in dialysis patients, with a lower pill burden and better adherence. We observed that former Sev patients switched to SO have a 62% and 59% decrease in prescribed PB pill burden at SO 1 – 3 and SO 4 – 6, respectively. These results support the findings of Floege et al. [9] who reported the mean daily SO pill number over the 28-week extension study to be lower than the number of pills for Sev (4.0 vs. 10.1 pills/day). 

When comparing baseline and follow-up periods, we observed a small but statistically significant reduction in epoetin-α use and dose, and a decline in the proportion of patients receiving IV iron. Although SO is an iron-based PB, studies have demonstrated minimal gastrointestinal absorption of iron from SO [8, 10]. Thus, it is not clear if SO contributed to the reduction in epoetin use and dose or if recent changes in policy and labeling have altered ESA practice patterns [11, 12]. 

Other noteworthy results of our analysis include generally stable levels of iron parameters among patients not treated with IV iron, as well as an increase of iron indices among patients on IV iron therapy. Observed changes are consistent with previously reported phase III study results by Floege et al. [8]. An increase in ferritin concentrations was observed at 6 months of follow-up among patients treated with IV iron. The otherwise unaltered levels of ferritin and transferrin among those not treated with IV iron support the low absorption of iron from SO. Covic et al. [13] reported results of a phase III study of SO and attributed observed changes in iron parameters mainly to concomitant IV iron use. 

We observed a small change in enPCR, a 0.02 g/kg/day decline from baseline during both SO time periods. The enPCR is an indirect measure of dietary protein intake. The KDOQI guidelines recommended daily protein intake for patients on HD is 1.2 g/kg/day [14]. Overall, unaffected serum albumin levels are in support of no major changes in dietary habits or decrease in daily protein intake. Dialysis adequacy, measured by eKt/V, remained unchanged. 

Several limitations of this database analysis must be noted. This is a retrospective observational study, and routine clinical data were extracted from existing electronic records and not collected for research purposes. As such, there are no data available on treatment indication, adherence, and tolerance. For instance, although medication delivery dates are known, when the patient actually stops taking one medication and begins taking the new medication is unknown. There were 605 patients included in the 1 – 3 month cohort that were ineligible for the 4 – 6 month cohort due to lack of serum phosphorus measurements, no PB prescription at FreseniusRx, switch to prescription of other PB or PBs, or prescription fills of SO and other PB. In addition, we have no direct information about patients’ nutritional habits and dietary phosphorus intake. This study lacks a concurrent control group and therefore cannot account for any temporal changes. Finally, this observational database analysis is limited by a fairly short, 6-month follow-up period. 

In conclusion, SO, a non-calcium, iron-based PB demonstrated control of serum phosphorus levels with a low mean prescribed number of pills per day. The effectiveness of SO was evident at three as well as at 6 months of follow-up, although 605 patients were not included for all follow-up. The mean number of prescribed PB pills was reduced from 9.7 to 4.0 pills/day (> 50% decrease) and the proportion of patients with serum phosphorus ≤ 5.5 mg/dL increased from 15.6 to 30.4% (95% increase) by the end of the 6-month follow-up. 

## Conflict of interest 

LHF, VP, LA, SV, NJO, CM, FWM, and RJK are employees of Fresenius Medical Care, North America. CM, FWM, and RJK own stock in the company. RJK is on the Board of Directors of Advanced Renal Technologies. DWC is a consultant and speaker for Fresenius Medical Care North America, and a consultant for GSK, Vifor, AMAG, Eli Lilly, and Medibeacon. SMS receives consultancy fees from OPKO Health, Vifor Pharma, Amgen, Fresenius Medical Care, and research funding from Abbott, Amgen, Cytochroma/OPKO Health, Vifor Pharma, and Deltanoid. 

## Acknowledgment 

We thank Carly Van Zandt for data extraction and de-identification. 

**Table 1.** Baseline characteristics for cohort of patients with 1 – 3 months (n = 1,029) and 4 – 6 months (n = 424) of SO prescription.

Baseline characteristics	Cohort with 1 – 3 months of SO prescription	Cohort with 4 – 6 months of SO prescription
Number of patients	1,029	424
Age, years	52.4 ± 13.0	51.2 ± 12.9
BMI, kg/m^2^	32.3 ± 8.8	32.3 ± 9.1
Time from first dialysis, months	53.3 ± 46.7	55.9 ± 49.1
Male, n (%)	555 (53.9)	240 (56.6)
Race, n (%)		
White	522 (50.7)	204 (48.1)
Black/African American	452 (43.9)	195 (46)
Other	55 (5.4)	25 (5.9)
Hispanic/Latino, n (%)	163 (15.8)	71 (16.8)
Comorbidities, n (%)		
Diabetes mellitus	605 (58.8)	246 (58)
Congestive heart failure	215 (20.9)	80 (18.9)
Reason for ESRD, n (%)		
Hypertension	338 (32.9)	143 (33.7)
Diabetes mellitus	428 (41.6)	171 (40.3)
Glomerulonephritis	43 (4.1)	15 (3.6)
Other	220 (21.4)	95 (22.4)
Baseline phosphate binder, n (%)		
Calcium acetate	242 (23.5)	94 (22.2)
Sevelamer	629 (61.1)	269 (63.4)
Lanthanum carbonate	72 (7)	32 (7.6)
Recorded switch between phosphate binders	86 (8.4)	29 (6.8)
Anemia therapy, n (%)		
IV iron use^a^	810 (78.7)	331 (78.1)
ESA use^b^	879 (84.6)	353 (83.3)

Values are expressed as mean ± SD or number (%) of patients. ^a^IV iron treatment includes iron sucrose, ferumoxytol, and sodium ferric gluconate; ^b^ESA treatment includes epoetin-α, epoetin-β and methoxy polyethylene glycol, and darbepoetin-α; BMI = body mass index; ESA = erythropoiesis stimulating agent; ESRD = end stage renal disease; IV = intravenous; SO = sucroferric oxyhydroxide.

**Table 2.** Comparison of changes in clinical parameters and anemia therapy prescription patterns between baseline and SO 1 – 3 for patients with 1 – 3 months of SO follow-up (n = 1,029).

Parameter	Baseline	SO 1 – 3	p-value
Pill burden			
Prescribed PB pills/day	9.6 ± 4.3	3.8 ± 1.3	< 0.001
MBD markers			
Patients with serum phosphorus ≤ 5.5 mg/dL, n (%)	143 (13.9)	269 (26.1)	< 0.001
Serum phosphorus, mg/dL	6.93 ± 1.39	6.65 ± 1.61	< 0.001
Serum calcium, mg/dL	9.24 ± 0.69	9.19 ± 0.72	0.002
iPTH, pg/ml	578.1 ± 479.8	598.4 ± 506.2	0.06
Clearance and nutritional parameters			
Albumin, g/dL	3.96 ± 0.31	3.96 ± 0.31	0.8
enPCR, g/kg/day	0.97 ± 0.21	0.95 ± 0.21	0.001
eKT/V	1.46 ± 0.22	1.46 ± 0.23	0.6
Patients receiving IV iron therapy (n = 895)^a^			
Iron indices			
Ferritin, ng/mL	986.0 ± 484.3	1014.3 ± 478.1	0.1
TSAT, %	34.1 ± 10.4	35.5 ± 12.3	0.001
Hemoglobin, g/dL	10.9 ± 1.0	10.9 ± 1.1	0.9
Anemia therapy medications			
IV iron use, n (%)^a^	810 (90.5)	720 (80.4)	< 0.001
Iron sucrose use, n (%)	808 (99.8)	718 (99.7)	< 0.001
Iron sucrose dose, mg/week	66.9 ± 42.9	71.0 ± 57.1	0.1
ESA use, n (%)^b^	773 (86.4)	738 (82.5)	< 0.001
Epoetin-α use, n (%)	735 (95.1)	679 (92)	< 0.001
Epoetin-α dose, U/HD Tx	3560 ± 3288	3534 ± 3608	0.8
Epoetin-α dose, U/administration	5225 ± 4322	4957 ± 4315	0.01
Patients not receiving IV iron therapy (n = 134)^a^			
Iron indices			
Ferritin, ng/mL	1151.9 ± 597.5	1113.1 ± 842.2	0.6
TSAT, %	38.0 ± 15.0	35.9 ± 15.2	0.06
Hemoglobin, g/dL	11.2 ± 1.5	11.2 ± 1.6	0.9
Anemia therapy medications			
ESA use, n (%)^b^	97 (72.4)	88 (65.7)	0.007
Epoetin-α use, n (%)	93 (95.9)	82 (93.2)	0.002
Epoetin-α dose, U/HD Tx	3389 ± 3411	3382 ± 3454	0.9
Epoetin-α dose, U/administration	5074 ± 4358	4892 ± 4462	0.5

Values are expressed as mean ± SD or number (%) of patients; ^a^IV iron treatment includes iron sucrose, ferumoxytol, and sodium ferric gluconate; ^b^ESA treatment includes epoetin-α, epoetin-β, and methoxy polyethylene glycol, and darbepoetin-α; ESA= erythropoiesis stimulating agent; iPTH = intact parathyroid hormone; IV = intravenous; MBD = mineral bone disease; PB = phosphate binder; enPCR = equilibrated normalized protein catabolic rate; SD = standard deviation; SO = sucroferric oxyhydroxide; TSAT = transferrin saturation; Tx = treatment.

**Table 3.** Comparison of changes in clinical parameters and anemia therapy prescription patterns between baseline and SO 4 – 6 for patients with 4 – 6 months of SO follow-up (n = 424).

Parameter	Baseline	SO 4-6	p-value
Pill burden			
Prescribed PB pills/day	9.7 ± 4.4	4.0 ± 1.4	< 0.001
MBD markers			
Patients with serum phosphorus ≤ 5.5 mg/dL, n (%)	66 (15.6)	129 (30.4)	< 0.001
Serum phosphorus, mg/dL	6.86 ± 1.44	6.41 ± 1.61	< 0.001
Serum calcium, mg/dL	9.24 ± 0.70	9.11 ± 0.72	< 0.001
iPTH, pg/mL	608.5 ± 524.9	652.7 ± 543.7	0.03
Clearance and nutritional parameters			
Albumin, g/dL	3.98 ± 0.30	3.97 ± 0.31	0.08
enPCR, g/kg/day	0.96 ± 0.2	0.94 ± 0.2	0.01
eKT/V	1.46 ± 0.21	1.45 ± 0.21	0.5
Patients receiving IV iron therapy (n = 393)^a^			
Iron indices			
Ferritin, ng/mL	984.1 ± 484.6	1,070.7 ± 510.1	< 0.001
TSAT, %	34.3 ± 11.1	35.7 ± 12.4	0.09
Hemoglobin, g/dL	10.9 ± 1.0	10.9 ± 1.1	0.5
Anemia therapy medications			
IV iron use, n (%)^a^	331 (84.2)	276 (70.2)	< 0.001
Iron sucrose use, n (%)	330 (99.7)	265 (96)	< 0.001
Iron sucrose dose, mg/week	67.8 ± 44.3	58.2 ± 39.5	0.02
ESA use, n (%)^b^	339 (86.3)	323 (82.2)	0.01
Epoetin-α use, n (%)	321 (94.7)	281 (87)	< 0.001
Epoetin-α dose, U/HD Tx	3,613 ± 3,330	3,182 ± 3,379	0.03
Epoetin-α dose, U/administration	5,397 ± 4,581	4,805 ± 4,116	0.02
Patients not receiving IV iron therapy (n = 31)^a^			
Iron indices			
Ferritin, ng/mL	1161.1 ± 673.8	926.5 ± 572.9	0.005
TSAT, %	38.3 ± 15.3	38.9 ± 16.0	0.7
Hemoglobin, g/dL	12.0 ± 1.7	12.1 ± 1.7	0.5
Anemia therapy medications			
ESA use, n (%)^b^	14 (45.2)	12 (38.7)	0.3
Epoetin-α use, n (%)	14 (100)	11 (91.7)	0.2
Epoetin-α dose, U/HD Tx	4,480 ± 4,813	3,851 ± 4,597	0.3
Epoetin-α dose, U/administration	5,689 ± 5,171	4,989 ± 4,600	0.4

Values are expressed as mean ± SD or number (%) of patients. ^a^IV iron treatment includes iron sucrose, ferumoxytol, and sodium ferric gluconate; ^b^ESA treatment includes epoetin-α, epoetin-β and methoxy polyethylene glycol, and darbepoetin-α; ESA= erythropoiesis stimulating agent; iPTH = intact parathyroid hormone; IV = intravenous; MBD = mineral bone disease; PB = phosphate binder; enPCR = equilibrated normalized protein catabolic rate; SD = standard deviation; SO = sucroferric oxyhydroxide; TSAT = transferrin saturation; Tx = treatment.

**Table 4.** Recorded phosphate binder therapy during 4 – 6 months in patients who switched from SO to other binders (n = 199).

Phosphate binder after switch	Number of patients
PB monotherapy	
Sevelamer	108
Calcium acetate	53
Lanthanum carbonate	13
Ferric citrate	2
PB dual therapy	
Calcium acetate + sevelamer	14
Calcium acetate + lanthanum carbonate	5
Lanthanum carbonate + sevelamer	3
Lanthanum carbonate + ferric citrate	1
Total	199

PB = phosphate binder; SO = sucroferric oxyhydrase.
